# Feature selection using a one dimensional naïve Bayes’ classifier increases the accuracy of support vector machine classification of CDR3 repertoires

**DOI:** 10.1093/bioinformatics/btw771

**Published:** 2017-01-05

**Authors:** Mattia Cinelli, , Yuxin Sun, Katharine Best, James M Heather, Shlomit Reich-Zeliger, Eric Shifrut, Nir Friedman, John Shawe-Taylor, Benny Chain

**Affiliations:** 1 Division of Infection and Immunity, UCL, London, UK; 2 Department of Computer Science, UCL, London, UK; 3 Complex, UCL, London, UK; 4 Department of Immunology, Weizmann Institute, Rehovot, Israel

## Abstract

**Motivation:**

Somatic DNA recombination, the hallmark of vertebrate adaptive immunity, has the potential to generate a vast diversity of antigen receptor sequences. How this diversity captures antigen specificity remains incompletely understood. In this study we use high throughput sequencing to compare the global changes in T cell receptor β chain complementarity determining region 3 (CDR3β) sequences following immunization with ovalbumin administered with complete Freund’s adjuvant (CFA) or CFA alone.

**Results:**

The CDR3β sequences were deconstructed into short stretches of overlapping contiguous amino acids. The motifs were ranked according to a one-dimensional Bayesian classifier score comparing their frequency in the repertoires of the two immunization classes. The top ranking motifs were selected and used to create feature vectors which were used to train a support vector machine. The support vector machine achieved high classification scores in a leave-one-out validation test reaching** **>90% in some cases.

**Summary:**

The study describes a novel two-stage classification strategy combining a one-dimensional Bayesian classifier with a support vector machine. Using this approach we demonstrate that the frequency of a small number of linear motifs three amino acids in length can accurately identify a CD4 T cell response to ovalbumin against a background response to the complex mixture of antigens which characterize Complete Freund’s Adjuvant.

**Availability and implementation:**

The sequence data is available at www.ncbi.nlm.nih.gov/sra/?term¼SRP075893. The Decombinator package is available at github.com/innate2adaptive/Decombinator. The R package e1071 is available at the CRAN repository https://cran.r-project.org/web/packages/e1071/index.html.

**Supplementary information:**

Supplementary data are available at *Bioinformatics* online.

## 1 Introduction

We have previously used short read parallel high-throughput sequencing (HTS) to estimate T cell receptor β transcript frequencies and sharing (Madi *et al.*, 2014; Ndifon *et al.*, 2012), and to explore the global changes in the CD4+ T cell receptor repertoire following immunization of mice (Thomas *et al.*, 2014). The latter study focused on local features of protein sequence within the TCRβ CDR3 loop, which interacts directly with peptide antigen lying within the MHC groove. The TCRβ CDR3 encodes the largest amount of sequence diversity, coded for by the combination of the ends of the V and J genes, D regions, and the DJ and VD junctions that include random nucleotide insertions. We therefore mapped the sets of TCRβ CDR3 sequences from each animal to a lower dimensional feature space indexed by short stretches of contiguous amino acids (typically triplets). Classical regularized machine learning algorithms (e.g. Support Vector Machines) were then able to distinguish between TCR repertoires of unimmunized mice and mice immunized with an extract of Mycobacterium tuberculosis (Complete Freund’s Adjuvant, CFA) within the lower dimensional transformed feature space. These studies suggested that short amino acid motifs within the TCRβ CDR3 region might contribute to defining TCR specificity.

CFA contains a complicated mixture of protein and non-protein antigens, and causes more widespread perturbations of the repertoire than single protein antigens. However, purified protein antigens are poorly immunogenic except when given in the context of adjuvants, which are believed to provide a danger signal which stimulates innate immunity and hence drives effective antigen presentation via T cell co-stimulation (Ibrahim *et al.*, 1995; Janeway, 1989). We therefore wished to extend our investigation to analyze the response to a well-studied model antigen, ovalbumin (OVA), when delivered in the context of CFA.

The strategy adopted previously to classify between unimmunized and immunized mice based on the frequency of short amino acid motifs could not effectively distinguish between mice given adjuvant with or without an additional protein antigen. This stems from the relatively small change in repertoire composition that is generated by a single antigen, which is not captured by our previous approach. However, introducing an additional prior step of feature selection using a 1-dimensional linear Bayes function in order to filter out noise, and further reduce dimensionality, proved successful. This significantly extends the generality of our previous finding by demonstrating that the frequency of a small number of linear motifs three amino acids in length can accurately identify a CD4 T cell response to ovalbumin against a background response to the complex mixture of antigens which are found in CFA. Small sets of conserved amino acid strings may contribute antigen specificity while allowing sufficient degeneracy to mount a robust immune response in all individuals even in the context of extreme sequence variability.

## 2 Materials and methods

*Sample collection and sequencing* 9 C57BL/6 mice were immunized with CFA, and 9 mice were immunized with an emulsion of CFA and OVA (Sigma, Poole, UK) in phosphate buffered saline (PBS) (100µg/mouse). After immunization, mice were sacrificed and spleens collected after 5, 14 or 60 days. Mice taken down at 60 days were given a booster of Incomplete Freund's Adjuvant (mineral oil) emulsified with or without OVA at day 14. CD4+ T cells were isolated from spleens and TCRβ chains from these cells were sequenced via the protocol described in (Ndifon *et al.*, 2012). Briefly, total RNA was reverse transcribed with a primer specific to the TCRβ constant region, and resulting cDNA was amplified via PCR using a set of TCRVβ primers. Illumina adaptors were ligated to the product, including indexes to identify each sample, and the sequencing was performed using Genome Analyzer II which generates forward and reverse 50 base pair reads. The sequence files are available at http://www.ncbi.nlm.nih.gov/sra/?term=SRP075893. The number of total and unique sequences from each repertoire is shown in Supplementary Data Figure S1.

*Data preprocessing* Raw sequence data was analyzed and error corrected using a short read modification of Decombinator as described in detail previously (Thomas *et al.*, 2014).

*Sequence features* Each TCRβ CDR3 sequence was mapped to a numeric string feature. The string feature is the number of times (term frequency) each p length substring (typically triplets, p = 3, number of features = 20^3^= 8000) appears in a set of TCRβ CDR3 sequences (i.e. a repertoire). In order to normalize for the size of the datasets from each mouse, 11 equal size sets of 100 000 TCRβ CDR3s were randomly selected from each mouse. The analysis was performed both on data in which multiple identical CDR3s were retained, and on data in which each distinct CDR3 was only counted once. Similar qualitative results were obtained, but the results shown below is on data in which relative abundance information was retained.

*One dimensional Bayesian classifier (1-DBC)*. 1-DBC is an application of Bayes’ rule to compute the ratio of the log probabilities of a feature belonging to either of two classes. The frequency of each feature in the two classes is modelled using Gaussian distributions based on estimates of the means and the standard deviations of the frequency with which the feature is found in each class.

$${DBayes}_{}\left(x\right)=\log\frac{P\left(C_{1}\right)}{P\left(C_{2}\right)}-\frac{1}{2}\left(\log\frac{\sigma_{1}^{2}}{\sigma_{2}^{2}}+\frac{1}{\sigma_{1}^{2}}{\left(x-\mu_{1}\right)}^{2}-\frac{1}{\sigma_{2}^{2}}{\left(x-\mu_{2}\right)}^{2}\right)$$

where P(C_i_) is the relative frequency of the feature x in the two populations, and µ_i_ and σ_i_ are the parameters of the Gaussians fitted for each class. If D_Bayes_ (x)> 0, feature x is classified into class C_1_, and if D_Bayes_ (x)<0, it is classified into class C_2_.

Using a leave-one-out validation strategy, we evaluated the classification accuracy of each amino acid motif in distinguishing the two classes of repertoire, those from mice immunized with CFA alone, or those from mice immunized with CFA and ovalbumin. This classification accuracy was used as a score with which to rank all the motifs.

*Support vector machines (SVM)*. SVM algorithms seek a linear hyperplane that separates observations from two (or more) distinct classes. The separating hyperplane is found such that the margin between the hyperplane and the nearest observations from the training data from each class is maximized, and the observations that define the size of the margin are termed support vectors, lending the method its name. Soft-margin optimization is carried out via the introduction of slack variables (Cortes and Vapnik, 1995) to determine the optimal hyperplane for non-linearly separable data. The data can also be transformed into a higher dimensional space, where linear separation may be possible, using the so-called kernel trick (reviewed in (Cristianini and Shawe-Taylor, 2000)). We have chosen SVM since it regularizes the weight vector minimizing a combination of its 2-norm with the chosen loss function (in this case the hinge loss). This ensures that SVM can perform well even when the feature space is very high dimensional. SVM were implemented using the R ‘e1071’ (Cristianini and Shawe-Taylor, 2000) package using linear or radial basis kernels.

## 3 Results

We analyzed TCRβ CDR3 sequences from a total of 18 mice, immunized with CFA emulsified with either PBS only (9 mice) or OVA dissolved in PBS (9 mice). Following from our previous work (Thomas *et al.*, 2014) we created feature vectors from the frequency of single amino acids (vector of length 20), duplets (all combinations of two consecutive amino acids, vector of length 400) or triplets (all combinations of three consecutive amino acids, vector of length 8000). SVM (we used both linear and radial basis kernels) were then trained on these feature vectors as described in (Thomas *et al.*, 2014). Since the number of samples available was small, we tested classification accuracy using a leave-one-out approach and combined all the time points. However, the classification efficiencies achieved in each case were poor (i.e. not much better than random). Similar poor results were achieved using frequencies of V or J regions, or a combination thereof.

**Fig. 1:**
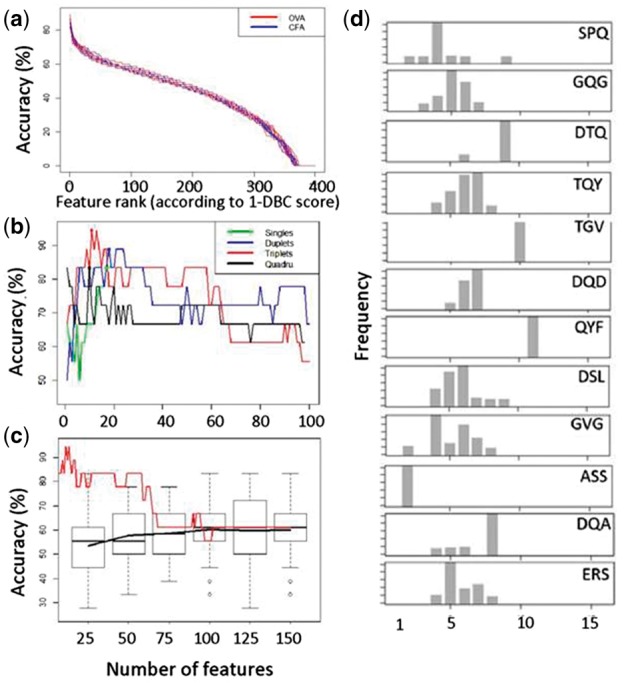
(**a**) The ranked 1-DBC classification efficiency for all amino acid duplets. Each line represents the trajectory for a different ‘outer’ leave-one-out selection. (**b**) Classification accuracy using an SVM trained on increasing numbers of p-tuples. The p-tuples were selected on the basis of decreasing classification accuracy in the 1-DBC. (**c**) Comparison of (b) to classification accuracy using an SVM trained on increasing numbers of randomly selected triplets (box and whiskers plot shows median, inter-quartile range and range for 100 different random samples). Solid black line shows means of random features. Red line shows the performance of triplets selected on the basis of decreasing classification accuracy in the 1-DBC (as in b). (**d**) The relative positional distribution of the top twelve ranked triplets (by 1-DBC classification score) along the CDR3. The histograms show the percent times that each triplet starts at that relative position, using a sample of TCRβ CDR3s from all repertoires combined. Since the TCRβ CDR3s are of different length, the starting position of each feature is calculated as a proportion of the CDR3 length

In order to improve SVM performance, we introduced a prior feature selection step to limit the number of features used, and to potentially reduce noise. In order to avoid any possibility of including training data at the test step we used a double leave-one-out strategy. One repertoire sample was first set aside as the eventual SVM test sample (we refer to this as the ‘outer’ leave-one-out repertoire). Then each remaining repertoire sample was excluded in turn (the ‘inner’ leave-one-out repertoire) and the remaining (n-2) samples were used to calculate the means and variance of the frequency of each p-tuplet in the two classes, CFA alone and CFA plus OVA. These parameters were used to calculate DBayes (x) for each p-tuplet and hence classify the ‘inner’ left-out repertoire in turn. The accuracy of the p-tuplet, tested for its ability to correctly classify all ‘inner’ leave-one-out repertoires, constituted the 1-DBC score. Since 11 subsamples were used from each repertoire, and each subsample created a slightly different set of feature scores, we averaged the scores of each feature across all subsamples. The ranked average 1-DBC score for p = 2 is plotted in Figure 1a. Similar results were obtained for p = 1, p = 3, and p = 4.

We then selected varying numbers of features in decreasing ranked order of 1-DBC score to train an SVM using all samples except the original ‘outer’ leave-one-out sample. Finally, this SVM was used to classify the original ‘outer’ test sample. The results for p = 1, 2, 3 and 4 are shown in Figure 1b. The qualitative pattern observed is the same for all size p-tuplets. The classification accuracy rises initially with increasing number of features. However, the accuracy quickly reaches a maximum, and then decreases as the number of features increases. The best overall accuracy is seen with triplets as observed previously (Thomas *et al.*, 2014), and further analysis focuses on these motifs.

The optimum number of features observed in Figure 1b is determined post facto and cannot therefore be used to determine the number of features to use a priori. In order to select the number of features to use independently of the classification results obtained, we took advantage of the pronounced elbow seen within the first 20 features in the feature score plots (Fig. 1a). We determined the position of this elbow by visual inspection, and then set the number of features used in the SVM to this value. The results obtained are shown in Table 1. In general, classification results for each repertoire were quite stable to sampling (i.e. classification accuracy was 0 or 100%). One repertoire was consistently misclassified irrespective of method used.

**Table 1.** The classification accuracy of combined 1-BDC and SVM on all repertoires analyzed^1^

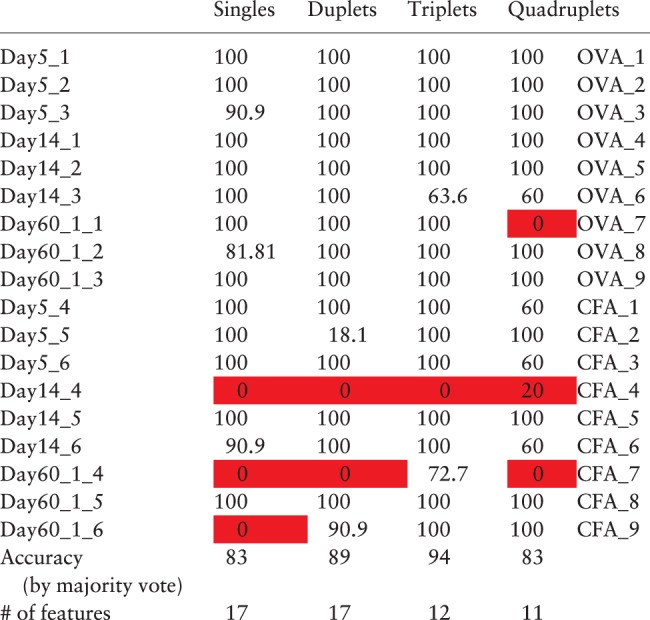

^1^The results of the SVM classifier using the top features ranked according to 1-DBC score. The number of features used is shown in the last row. Each row shows the % correct classification for one left-out repertoire, using 11 samples of 100 000 TCRβ CDR3s from that repertoire as test (solid background indicates misclassified cases). The penultimate row shows the overall classification efficiency, where the classification of each mouse is made by majority vote.

We wanted to determine whether the increased classification accuracy we observed was simply a function of using a smaller number of features, or whether the 1-DBC score in fact selected more informative features. We therefore compared the classification efficiency observed using different numbers of features selected by their 1-DBC score, with the results obtained using 100 repeated random samples of the same number of features. As shown in Figure 1c (and Supplementary Data Fig. S2), using the features selected by 1-DBC score reached >90% accuracy, outperforming the random sets of features for small numbers of features (up to about 50). For a larger number of features accuracy declines to the average accuracy observed using randomly selected features, which was around 60%.

We wished to compare the performance of our repertoire classification with previous published methods. To our knowledge, our previous publication (Thomas *et al.*, 2014) is the only one which seeks to classify TCR repertoires directly. Using the methods used in this previous study (initial clustering of triplets using Kidera factors, followed by an SVM) and the same data as analyzed in Table 1, we obtained an accuracy of 77% (cf 94% in Table1). An SVM using all 8000 triplet features (i.e. with no preclustering) performed worse, with an accuracy of 67%. Finally, we used Random Forest classification, which has been shown to perform well on high dimensional datasets where overfitting is often a problem. Using the R package randomForest we obtained a maximum accuracy of 67%. Thus the combination of feature selection and SVM outperforms several current state-of-the-art high dimensional classification methods on this dataset.

The twelve triplets with the maximum 1-DBC score are examined further in Figure 1d. Since the CDR3s are of different length, the starting position of each feature is calculated as a proportion of the CDR3 length. Four of the 12 triplets were found predominantly at the beginning or end of the TCRβ CDR3 sequence, while 8 out of 12 of the triplets were found in the central region of the TCRβ CDR3. The 4 triplets at the extremes of the CDR3s shared sequence identity with the ends of germline V or J genes. However, no individual V or J genes were enriched in OVA or CFA repertoires (Supplementary Data Fig. S3).

## 4. Discussion

The computational pipeline described above tackles a challenging task, namely to distinguish between the global TCR repertoires of mice immunized with CFA alone and CFA plus OVA. CFA contains many different proteins, and contains a large number of possible T cell epitopes. In contrast, ovalbumin is a single protein which contains at most two or three I-A restricted CD4 T cell epitopes (Shimonkevitz *et al.*, 1983). Furthermore, the enormous size of the potential T cell repertoire and the complex non-germ line mutational process which creates this repertoire means that even genetically identical mice will contain largely disparate sets of receptors (Madi *et al.*, 2014; Murugan *et al.*, 2012). Despite this the pipeline was able to demonstrate good classification accuracy, by considering not individual TCRs or TCRβ CDR3s, but by analyzing the frequency of very short amino acid motifs or even the usage of individual amino acids within the repertoire taken as a whole.

The key to improving classification accuracy was the introduction of a prior feature selection step, before employing a more classical high dimensional classification tool such as SVM. The SVM algorithm contains a 2-norm regularization element, which in theory should limit the overfitting due to the high dimensionality of the feature sets used. The SVM alone, however, gave poor performance. The Bayes classifier used on single features similarly could not accurately classify the repertoires, since individual features provide very weak learners, and makes the assumption of a Gaussian distribution for feature frequencies. Nevertheless the classifier works well as a kind of filter, separating ‘useful’ from ‘noisy’ features. Prior feature selection presumably reduces the noise and forces the SVM classifier to focus on those features with the maximum information content.

In agreement with our previous study, triplets out-performed both shorter (singlets or duplets) or longer (quadruplets) features. Interestingly, some triplets with the highest scores were predominantly located towards the C or N terminals of the TCRβ CDR3 loops. These regions are, at least in part, often coded by the ends of the genomic V or J sequences that survived exonucleolytic processes during recombination. This observation is therefore in agreement with many reports that specific V regions are amplified selectively in certain antigen specific responses e.g. (Davis *et al.*, 1995; Kedzierska *et al.*, 2004). However, at least in this model, the frequencies of V and J alone were not sufficient for classification. Thus more complex parameters, such as combinations of V and J usage or convergent evolution of CDR3 sequences (Madi *et al.*, 2014; Miles *et al.*, 2011; Quigley *et al.*, 2010) must contribute to antigen specificity.

Our results demonstrate that specific antigen immunization, even in the context of co-exposure to complex other antigen mixtures, gives rise to changes in TCR repertoire which are coherent, conserved and recognizable. The success of classification methods using fairly simple low level features of protein sequence offer hopeful indications for applying this sort of approach to analysis of clinical samples for the prognosis, diagnosis or stratification of patients in the context of both infectious and non-infectious (e.g. cancer, autoimmunity, transplantation) disease.

## Supplementary Material

Supplementary Data
