# Supplementary material for: Feature selection using a one dimensional naïve Bayes’ classifier increases the accuracy of support vector machine classification of CDR3 repertoires
Source: Bioinformatics. 2017 Jan 5;33(7):951–5. doi: 10.1093/bioinformatics/btw771 (PMC5860388; doi:10.1093/bioinformatics/btw771)
Supplement: Supplementary Data [file btw771_supp.pptx]

## Slide 1
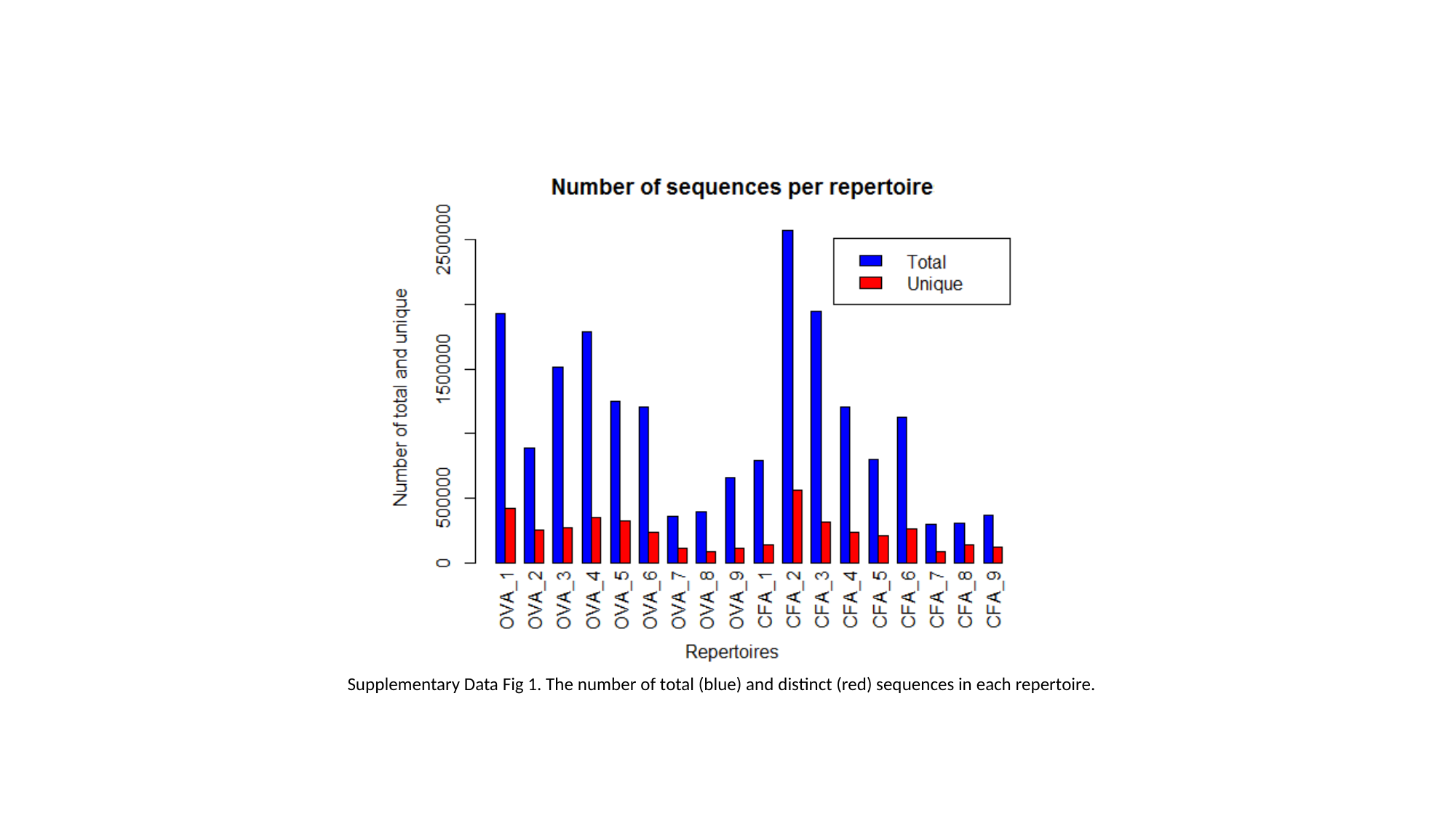

Supplementary Data Fig 1. The number of total (blue) and distinct (red) sequences in each repertoire.

## Slide 2
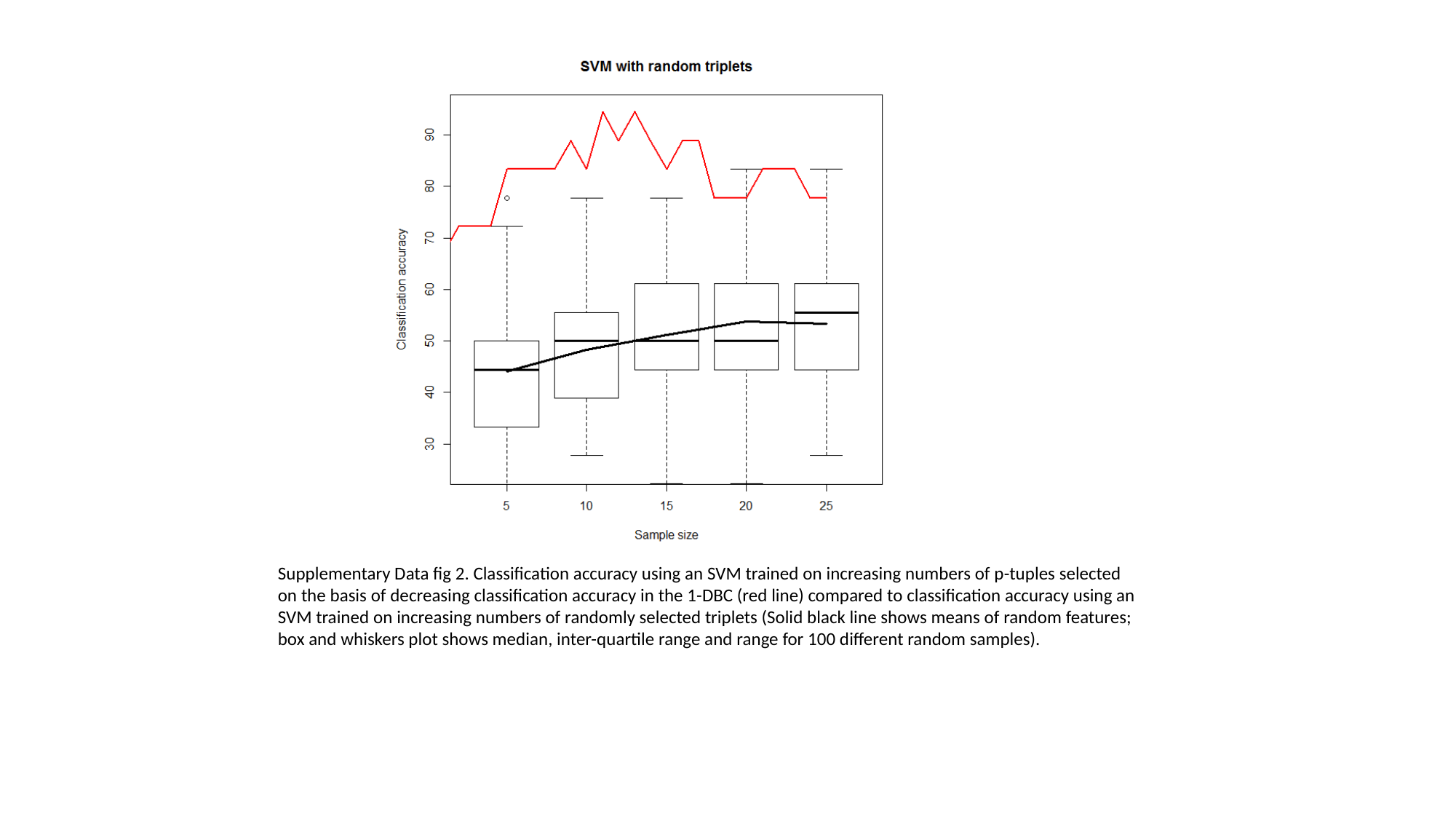

Supplementary Data fig 2. Classification accuracy using an SVM trained on increasing numbers of p-tuples selected on the basis of decreasing classification accuracy in the 1-DBC (red line) compared to classification accuracy using an SVM trained on increasing numbers of randomly selected triplets (Solid black line shows means of random features; box and whiskers plot shows median, inter-quartile range and range for 100 different random samples).

## Slide 3
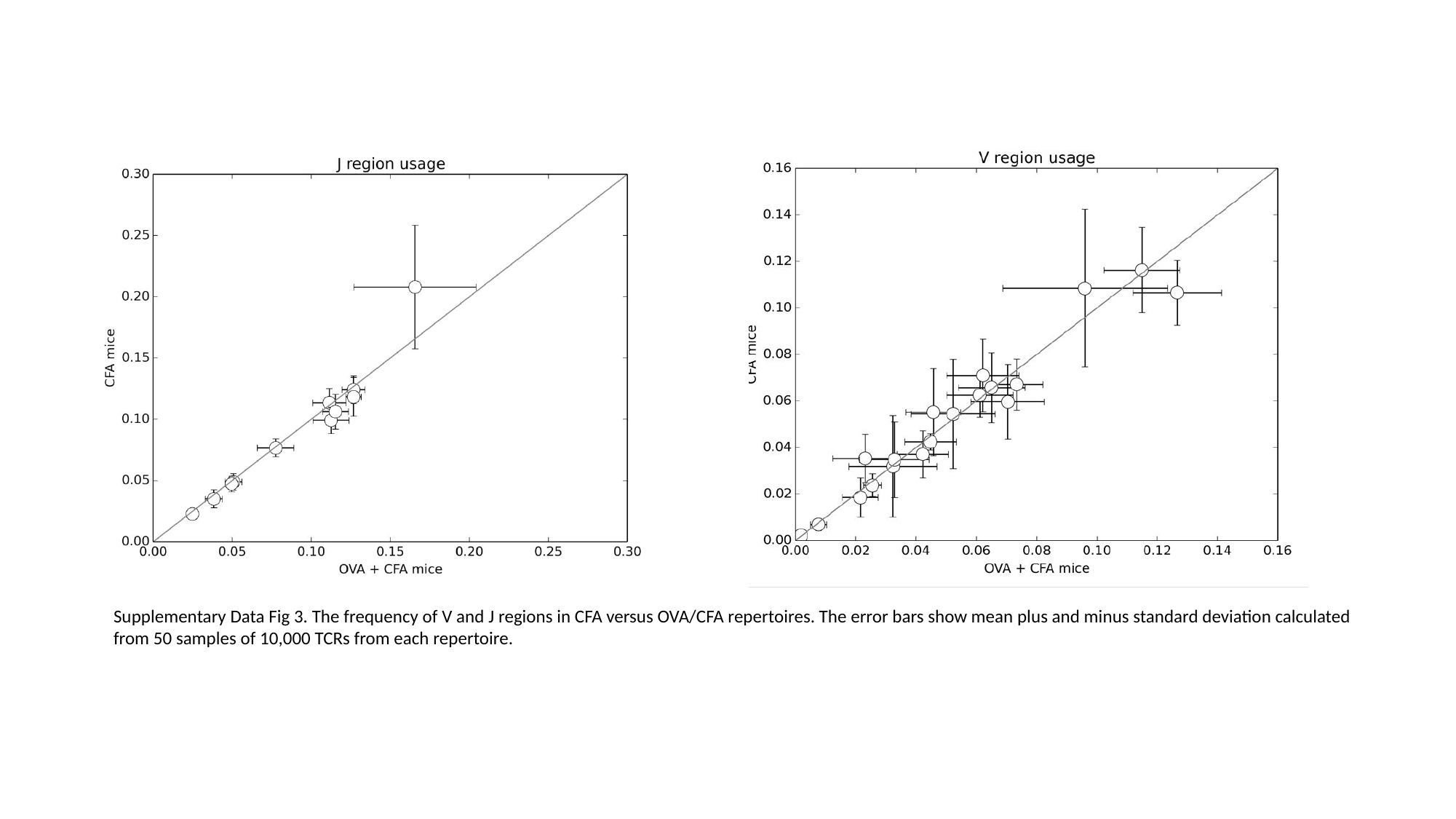

Supplementary Data Fig 3. The frequency of V and J regions in CFA versus OVA/CFA repertoires. The error bars show mean plus and minus standard deviation calculated from 50 samples of 10,000 TCRs from each repertoire.
